# Micro–Macro: Selective Integration of Microfeatures Inside Low-Cost Macromolds for PDMS Microfluidics Fabrication

**DOI:** 10.3390/mi10090576

**Published:** 2019-08-30

**Authors:** Edgar Jiménez-Díaz, Mariel Cano-Jorge, Diego Zamarrón-Hernández, Lucia Cabriales, Francisco Páez-Larios, Aarón Cruz-Ramírez, Genaro Vázquez-Victorio, Tatiana Fiordelisio, Mathieu Hautefeuille

**Affiliations:** 1 Facultad de Ciencias, Universidad Nacional Autónoma de México, CDMX 04510, Mexico; 2 Posgrado en Ciencias Biomédicas, Universidad Nacional Autónoma de México, CDMX 04510, Mexico; 3 Laboratorio Nacional de Soluciones Biomiméticas para Diagnóstico y Terapia (LaNSBioDyT), Universidad Nacional Autónoma de México, CDMX 04510, Mexico

**Keywords:** microfabrication, soft lithography, microfluidics

## Abstract

Microfluidics has become a very promising technology in recent years, due to its great potential to revolutionize life-science solutions. Generic microfabrication processes have been progressively made available to academic laboratories thanks to cost-effective soft-lithography techniques and enabled important progress in applications like lab-on-chip platforms using rapid- prototyping. However, micron-sized features are required in most designs, especially in biomimetic cell culture platforms, imposing elevated costs of production associated with lithography and limiting the use of such devices. In most cases, however, only a small portion of the structures require high-resolution and cost may be decreased. In this work, we present a replica-molding method separating the fabrication steps of low (macro) and high (micro) resolutions and then merging the two scales in a single chip. The method consists of fabricating the largest possible area in inexpensive macromolds using simple techniques such as plastics micromilling, laser microfabrication, or even by shrinking printed polystyrene sheets. The microfeatures were made on a separated mold or onto existing macromolds using photolithography or 2-photon lithography. By limiting the expensive area to the essential, the time and cost of fabrication can be reduced. Polydimethylsiloxane (PDMS) microfluidic chips were successfully fabricated from the constructed molds and tested to validate our micro–macro method.

## 1. Introduction

Microtechnology has contributed greatly to the progress of society thanks to the incredible growth of electronics that it has permitted. Photolithography has been the central process enabling fast miniaturization of individual components and complex layouts with metal, oxides and semiconductors required in circuit integration. Although the cost of materials and infrastructure required by this technology has always been relatively elevated, the high integration levels enabled by this technology have decreased the overall cost per chip. This said, when different construction materials such as polymers are required, or if small-volume rapid-prototyping devices are sought, microtechnology remains too expensive for many laboratories, especially in low-resource universities or developing countries. After the development of a more accessible method was introduced mainly thanks to the work of George Whitesides in soft-lithography [1,2], the fabrication of on-demand microchips has now become very popular, because it is more accessible. The techniques used in modern microtechnology have been greatly simplified and the catalog of compatible materials expanded significantly, hence broadening the range of possible applications [1,3,4]. The capability to pattern many materials at the microscale in a low-resource laboratory, even at a small production volume has increased the potential of prototyping and testing lab-on-chip technology, especially in biomedical applications where polymers in general, and poly-dimethylsiloxane (PDMS) in particular, are often used to guarantee a better transition from the laboratory to the market [5,6,7,8]. However, a serious limiting parameter in developing biomicrofluidic platforms is the geometric resolution [9]; the tools required to yield resolutions below 50 µm are still expensive [10,11]. One critical step in microfabrication using soft lithography, for instance, is the inevitable need for high-resolution micromolds used in replica-molding (REM) to reproduce the micropatterns with high fidelity in polymer materials such as PDMS. When high resolution is required, photolithographic processes are thus still used in order to obtain the master molds, although large low-resolution areas (such as those of the interconnection pads) do not necessarily need expensive patterning tools. Indeed, the cost associated with fabricating large areas on resist patterns transferred onto the mold substrates for subsequent replicas in PDMS is not ideal, as these common structures shared by all microfluidic chips usually do not require high-resolution processes with a very high associated cost per area. and different strategies may be employed nowadays [12]. 

Several simple solutions exist to create molds of direct fabrication: the use of thermoshrinkable polymers, the desktop cutter plotter or the combination of both are examples of these affordable techniques [10,13,14]. In our group, we have also developed a low-cost laser microfabrication system that also enables the construction of plastic micromolds [15]. This process appears to solve a cost issue when high-resolution features need to be integrated inside PDMS microfluidic chips using soft- lithography and rapid-prototyping. Indeed, usually photoresists are used to transfer patterns onto a wafer or a glass slide using a photomask, that also needs to be fabricated and usually presents a high associated cost that is not suitable for testing and prototyping. Other maskless techniques such as 2-photon lithography also guarantee high resolutions but their cost per area and processing time are too high for large surfaces (Table 1). Finally, the low-cost techniques widely used in low-resource laboratories are somehow limited to low-resolution features (Table 1). Therefore, we have decided to merge micro- and macro-fabrication procedures to restrict the area of high-resolution features fabricated by 2-photon lithography to the absolute minimum and hence greatly limit the cost and time of fabrication. Although large-scale structures have been manufactured using the 2-photon technique, there are still area limits and there is a need to use micromanipulators to make the connections with tubings and external pumps, which with our method may not be necessary [16].

In this work, we proposed to limit the area of micron-scale features to the absolute minimum in order to save process time and budget and hence present a standard procedure for the construction of microstructured PDMS microfluidic platforms useful for a low-resource laboratory or in educational laboratories. It is based on the separated design and fabrication of large areas of low- resolution structures for user-friendly interconnects as well as the high-resolution features but restricted to a smaller area, in a different step. This process allowed us a more efficient use of resources by producing the inlets/outlets layouts and larger channels of our chips in a very simple fashion, hence lowering the overall cost of the molds for the rapid prototyping of PDMS chips. It is now possible to design a “one-design-fits-all” macromold with interconnect layouts that may be readily made using simple techniques while micron-scale features are designed and fabricated apart and limited to the smallest area possible. Both molds are then replicated in PDMS using soft lithography and then bonded together to construct a single chip that integrates both micro- and macro-features. The ports (inlets and outlets) can be designed as a unique generic footprint with *n* available openings shared between multiple common microchannel designs. The openings may be designed sufficiently large in order to be compatible with any microfluidics connector (luers, reservoirs, micropipette tips, syringes, etc.).

Generic macro-layouts can thus be readily made in any laboratory using one of the multiple macromold techniques presented in our process, and it is then possible to outsource only a delimited area for the high-resolution micropatterns. The macromold fabrication process was validated here with several techniques such as plastics micromilling, laser microfabrication of plastics, or fabrication of molds using commercial shrinkable polystyrene sheets (Shrinky Dinks®, Alex Toys Inc., Fairfield, NJ, USA) [10,11]. The micron-range features were made using photolithography or 2-photon lithography, limiting the overall area to the essential in order to lower the cost. These high-resolution patterns were transferred either on a different mold or directly onto the macromold when possible for obtaining a single mold. In both micro and macro designs, special attention was drawn to the correct alignment of interconnections between both molds for the micro–macro process to be successful in the fabrication of microfluidics platforms. In order to validate the proof-of-concept, different chips were fabricated and are shown in this paper. Other similar methods have been proposed [17,18,19] but it is the combination of low-cost methods and the ability to create three-dimensional structures that make our work innovative.

## 2. Materials and Methods

### 2.1. Micro-Macro Integration Processes

**Figure 1:**
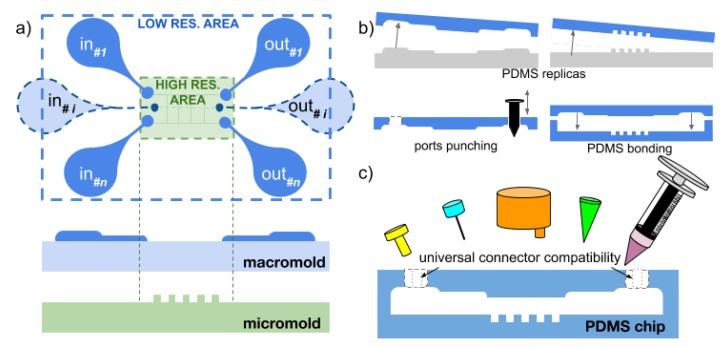
General principle of the Micro–macro process. (**a**) interconnect layouts are made in a macromold using low-resolution techniques while micron-scale features are designed and fabricated apart; (**b**) both molds are then replicated in polydimethylsiloxane (PDMS) and bonded together to create a single chip integrating both micro- and macro-features; (**c**) the ports (inlets and outlets) can be designed as a unique footprint with *n* available openings shared between multiple common microchannel designs. The openings may be designed sufficiently large in order to be compatible with any microfluidics connector (luers, reservoirs, micropipette tips, syringes, etc.).

**Figure 2:**
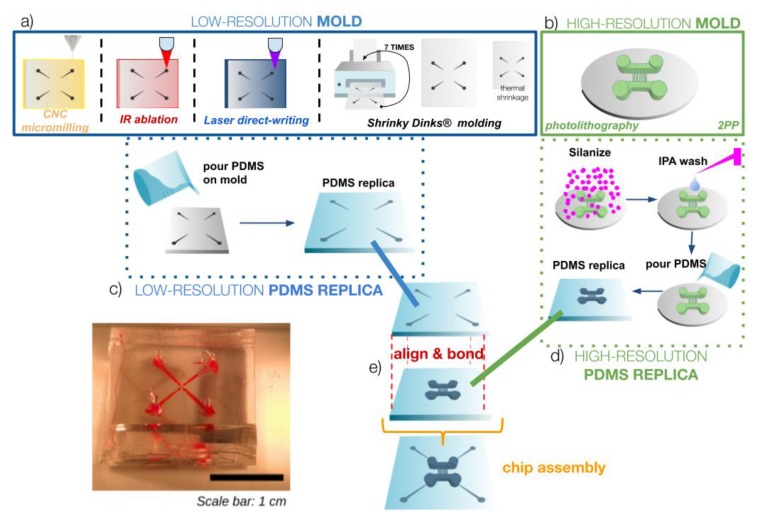
Micro–macro chip fabrication process using soft-lithographic replica-molding (REM) technique with 2 master molds. (**a**) A low-resolution mold of the larger features is developed with one of the multiple fabrication options: Computer Numerical Control (CNC) micromilling, laser ablation, laser direct-writing or polystyrene sheets molding (the pattern is printed 7 times on the same polystyrene (PS) sheet to add relief). (**b**) In parallel, a high-resolution mold is developed with one of the multiple options (photolithography, 2-photon polymerization or laser micromachining). (**c,d**) Then, two PDMS replicas are made using the REM technique. (**e**) Replicas are activated, aligned and bonded to each other to assemble the final chip, connecting micro features with macro patterns.

**Figure 3:**
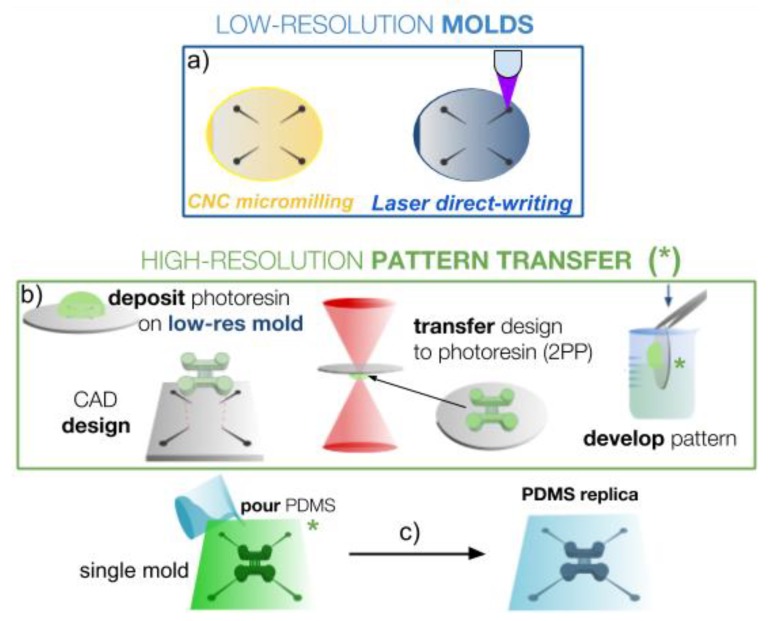
Micro–macro process using a single mold with both low-resolution and high resolution pattern transfer. (**a**) First, a low-resolution mold is made with CNC micromilling or laser-direct writing on a substrate compatible with the 2-photon laser polymerization (2PP) process. (**b**) Then a computer-aided design (CAD) containing microfeatures is transferred directly to the previous mold by 2PP to a compatible photoresin, developed and rinsed. (**c**) Finally, the single mold is used for the REM technique to obtain the final PDMS replica with macro- and micro-patterns on it.

The micro–macro process used in this work is aimed at integrating high-resolution (micro) patterns readily inside a larger low-resolution (macro) area in the design and fabrication of master molds in order to fabricate microfluidics chips with both large structures or interconnections and micro-sized features easily and almost directly for rapid-prototyping applications. The general process is presented in Figure 1 and the diagrams of Figure 2 and Figure 3 present the detailed procedures that were tested successfully in this work for two molds or a single mold respectively.

In the 2-mold option, the critical step was the alignment of the areas where the two layers of the chips had to be united correctly in order to ensure the flow from larger to smaller cross-sections (Figure 2). In the single-mold option, this step could be cancelled as the micro-features were directly fabricated on a pre-existing low-resolution mold, but particular attention was paid in precisely focusing the laser on the correct area to ensure continuity between large and small channels in the final chips. Different low-resolution macromolds were designed and fabricated with larger interconnects for inlet and outlet areas on the outer regions and smaller-size areas for interconnects with the high-resolution micro-features. The outer interconnects were made large to punch vias and holes compatible with all typical microfluidics interconnections depending on the application (luers, syringe needles, micropipette tips and commercial reservoirs). Both the low-resolution and high- resolution patterns of interest were fabricated using different techniques that are shown in Figure 2 and Figure 3 and detailed in the following sections.

For low-resolution mold fabrication, any of the following techniques was successfully employed: Computer Numerical Control (CNC) micromilling in an acrylic sheet, infrared laser ablation in an acrylic sheet, laser direct-writing to pattern a photoresin on a glass substrate, or shrinkable polystyrene sheet molding. In the case of the high-resolution mold, we used mask photolithography and 2-photon laser polymerization (2PP) of a photoresin. Each mold is then used in a replica-molding (REM) technique to obtain a high-fidelity PDMS replica of each mold, as described in detail in a following section. Then, both replicas are activated using plasma, UV-ozone, or corona discharge to promote PDMS–PDMS bonding. They are finally aligned carefully (using alignment marks that may be specifically designed, if necessary) under a stereoscopic microscope in order to assemble the final chip with interconnections easily accessible. The correct alignment of the micro- and macro-parts of the chip and absence of leaky joints is then verified by flowing a colored liquid inside the chip and visualizing the flow under a microscope.

In the case of the micro–macro single-mold process (Figure 3), only one substrate is used to obtain a single PDMS replica with both micro- and macro-features on them. The process is more sequential, as the high-resolution pattern is directly transferred onto the low-resolution mold using 2PP. In this case, only two of the four low-resolution techniques presented above were successfully used, due to two additional requirements of the 2PP technique. First, the substrate material needs to be compatible with the 2PP photoresin development process as the developer chemicals may be aggressive. Then, and more importantly, a smooth and flat substrate surface with a high enough refractive index is needed on the macromold to guarantee precise laser focusing and interface finding. Unfortunately, this high surface quality was not achieved in our laboratory with the other techniques and we thus restricted the low-resolution mold to CNC micromilling and laser direct-writing. Once these requirements were fulfilled, once the macromold was obtained, the substrate was inserted inside the 2PP laser system in order to transfer the desired CAD design onto the macromold. After development, the final mold contained both micro- and macro-features for subsequent REM and chip assembly processes.

### 2.2. Fabrication of Low-Resolution Macromolds

In this work, we tested four different techniques to fabricate the low-resolution or “macro” molds, accessible to most laboratories and relatively simple to implement rapidly. The molds obtained with these techniques consisted basically of substrates with larger structural parts or fluidic interconnect access ports of the final chip platforms. The exact procedures used in this work are detailed in the following.

#### 2.2.1. Low-Cost Shrinky Dinks® Molding

The use of commercial polystyrene (PS) sheets known as Shrinky Dinks® to construct 3D microfluidics channels very rapidly and at an affordable cost was first reported in 2008 [11] and our technique was based on this protocol. As mentioned in the literature, this technique offers great reproducibility from design to design, operator to operator and run to run. However, it is limited to structures with relatively low aspect ratios and usually presents a relatively high roughness that limits its use to low-resolution molds. Here, the desired macro pattern was designed in 2D using any design software and then printed 7 times at the same position of the PS sheet employing a 600 dpi commercial printer (Laser Jet CP1025nw, Hewlett-Packard, Palo Alto, CA, USA). After washing the surface carefully with a solution of 5% v/v of acetone in isopropyl alcohol (IPA) for several minutes in order to obtain well-defined boundaries, the polymer sheets were placed in a convection oven with homogeneous heating at 175 °C for 5 min until the shrinkage occurred. The samples were cooled down at ambient temperature for several minutes and stored hermetically until further use. The resolution of the 2.5D features of this kind of mold depends mainly on the quality of the printer and in this work a resolution of 200 µm was obtained for the macro-molds.

#### 2.2.2. Low-Cost Infrared Laser Ablation

Another low-cost and rapid technique used here for the low-resolution molds consisted of poly- methylmetacrylate (PMMA) laser ablation with a custom-made laser platform based on a motorized CD–DVD pickup head unit. The detailed procedure employed here was described in a previous work [15,20]. In this work, PMMA used as the laser platform offered excellent reproducibility and control of etched dimensions, including depth, over relatively large areas [15]. However, this process still was not suitable for applications where high resolution features of less than 20 µm are required.

#### 2.2.3. Low-Cost Blu-Ray Laser Direct Writing 

Similarly to IR laser ablation, a blu-ray pickup head unit was mounted on the previous platform to crosslink photosensitive resins [15]. In this work, we used a commercial UV-sensitive glue, Loctite 3525 (Henkel Corp., Düsseldorf, NRW, Germany), to construct macro-molds, using the procedure detailed in a previous work [15]. Simple 3D structures were readily made with this setup and technique, but the final resolution of the structures after development was not acceptable for high- resolution applications and its use was limited to the fabrication of low-resolution molds. 

#### 2.2.4. CNC Micromilling

The last option that was tested and presented excellent results to build macro-molds was CNC micromilling [21,22,23]. This subtractive manufacture technique is used to etch micrometric size patterns by means of a 3-axis motorized high-speed rotating cutting tool that removes bulk material. The precise position of this tool is controlled by Computer Numerical Control (CNC) via G-Code. In this work, we used a Mini-Mill/4 (Minitech Machinery Corp., Norcross, GA, USA) with up to 10 µm of resolution in each axis. The G-Code was generated with computer-aided design (CAD) software Fusion 360 (Autodesk Inc., San Rafael, CA, USA), which allows the straightforward translation of a 3D CAD model into a computer-aided manufacturing (CAM) cutting operation. The workflow followed here is a common CNC micromilling procedure, described as follows. First, the desired 3D structures were designed in CAD. In the case of the single-mold procedure where the CNC mold is then processed in 2PP, the CAD design integrated two separated bodies, both the micro- and the macro-features. In the macro-design, one or multiple alignment marks were added without affecting the function of the structures. In a second step, the G-Code instructions were created in order to engrave the structures correctly. Indeed, in this CAM, all manufacturing considerations that have to be followed in order to achieve optimum results were integrated: the size of the endmill, its feed rate, and the spindle speed were carefully selected after a proper characterization (presented in a following section) in order to achieve a low roughness surface in the lowest time possible. Another important factor in fabricating a mold for REM is the cutting strategy to guarantee a flat, even surface and homogeneous height across features. Because of its excellent performance for soft lithography REM using PDMS and its compatibility with the 2PP high resolutions process, in this work we used PMMA sheets (75 mm × 25 mm × 2 mm) to fabricate the macro-molds. To hold the polymer samples in place and guarantee a correct transfer, we used double sided tape [21]. 

### 2.3. Fabrication of Inner High-Resolution Micromolds

High-resolution features are usually expensive. Although our process is compatible with any high-resolution technique to fabricate molds, in this work the microstructures were fabricated using 2-photon polymerization of photosensitive resins. A Photonic Professional GT (PPGT) system from Nanoscribe GmbH (Eggenstein-Leopoldshafen, BW, Germany) was used with one of its proprietary resists (IP-S). Although the process enables a rapid, fully-automated transfer of a 3D pattern on compatible substrates from a simple CAD design, it is usually limited to relatively small areas for cost reasons. Moreover, in spite of some studies reporting the possibility to replicate the molds into PDMS layers, we found that the substrates typically require special treatment with silanes compounds for replication: first, adhesion promoters were needed on the clean substrates for the resin to attach and then fluorinated silanes had to be evaporated on the developed structures in order to avoid PDMS– resin adhesion when detaching in the REM process. In particular, we found that very small high-resolution patterns usually remained inside PDMS. Because the cost and risk of destructing the 2PP molds were high, we decided to limit the 2PP area to its absolute minimum, as presented in Figure 2 and Figure 3. In both cases of single or two-mold options, special attention was paid on the end- to-end connection area (low-resolution to high-resolution channels merging). A correct overlap was required to avoid possible leakage at high pressure when superficial tension needs to be broken for the flow to take place. In the case of the two-mold method, the connection area of both molds had enough tolerance to allow for a small misalignment. For the one mold method, visible alignment marks were designed and fabricated, easy to identify under the PPGT microscope to avoid any misalignment between the low and high-resolution processes. The discrepancy of the 2PP system is evaluated every 6 months in our laboratory and there is an average maximum difference of 0.35 µm between design and fabricated structures. This is below the 10% margin of permissible tolerance in our laboratory for the structures reported in this work.

### 2.4. Fabrication of Soft-Lithographic Replicas

The soft lithography technique of replica-molding (REM) was used to replicate the micro- and macro-molds in PDMS. This method consists in curing the elastomer on top of the master mold and transferring a negative copy of the geometries and structures of the mold into PDMS [1]. PDMS is the most common candidate to perform REM due to its interesting characteristics such as biocompatibility, non-cytotoxicity, simple surface treatment, and optical transparency, useful for microfluidics, microscopy, and cell culture. We used Sylgard® 184 Silicone Elastomer kit (Dow Corning, Midland, MI, USA) in a 10:1 w:w proportion of prepolymer and curing agent. The prepolymer and curing agent were mixed with a rotating tool at a constant speed for 5 min to ensure the homogeneity of the mixture. The mixture was then placed in a vacuum desiccator to remove bubbles formed during mixing. To ensure the PDMS replica detaching of the different molds, three methods were used to avoid adherence between the polymer and the substrate and detachment control (detailed below): no surface treatment in case of the low-resolutions molds, and evaporation of dichlorodimethylsilane (DMDCS) or trichloro(1H,1H,2H,2H-perfluorooctyl)silane (PFOCTS) (Sigma Aldrich, San Luis, MO, USA) in case of high-resolution molds. Indeed, the low-resolution molds did not require any surface treatment to obtain PDMS replicas. The molds were introduced in an aluminum- foil container. Then, the degassed PDMS was emptied into the container with the mold taking care not to create new bubbles between the mold and the PDMS interface. Immediately, the samples were placed in a convection oven preheated at 60 °C for 48 h. Although greater temperatures may be used to cure PDMS, it has to be avoided with the polystyrene sheets as the ink would melt and the mold would be destroyed. Finally, after the polymerization of the PDMS, the replicas were allowed to cool down so that the detachment of PDMS replica could be easily achieved. For the molds that used photoresins such as those made by laser direct-writing, or the high-resolution molds fabricated with photolithography, the substrates with negative structures had to have been previously silanized with DMDCS by evaporation. The molds were placed in an airtight container with a droplet of 1 mL of DMDCS for one hour at room temperature in an extraction hood. All the residual chemical vapors were finally allowed to evaporate by opening the container for 20 min at the end. 

For the molds printed using 2PP, a 10 µL droplet of PFOCTS was used and in this case the mold and the silane droplet were placed together, 1 cm apart, in a vacuum chamber for 2 h. When replicating high-resolution micro-molds, it was found that submerging the mold/replica in IPA helped reducing the risk of breaking or detaching the mold microstructures. Indeed, we had to use an IPA wash in order to eliminate the silane excess (manifesting itself as microscopic droplets) that only appears on 2PP high-resolution molds, probably caused by the reaction with the photocured Nanoscribe proprietary resins or with the substrate; generally, the 2PP photolithography is made on an ITO (Indium Tin Oxide) surface. The PFOCTS and DMDCS create Si–O covalent bonds with the oxygen at the surface, responsible for the droplets.

### 2.5. Microfluidic Interconnection

**Figure 4:**
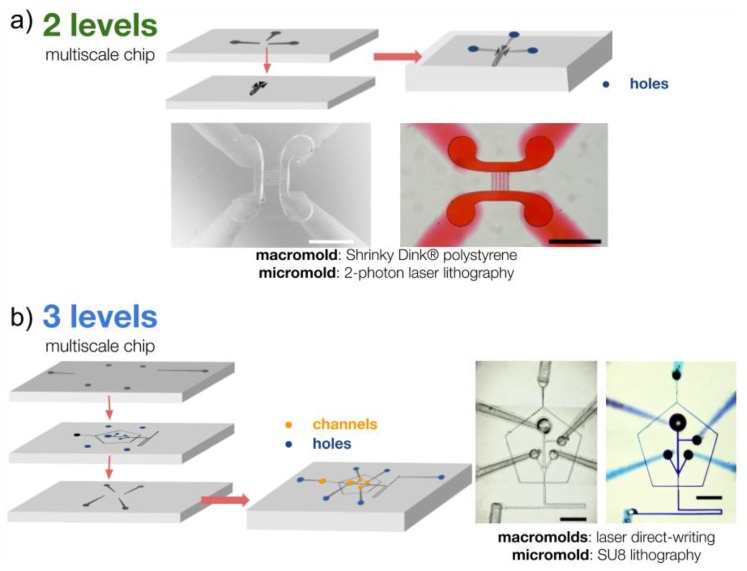
Examples of common microfluidic chips and platforms fabricated using our micro–macro process with multiple molds. (**a**) 2-level chips with one low-resolution mold and one high-resolution mold replicated in PDMS and bonded together aligning the channels interconnects before testing the flows. (**b**) Microdroplets chip fabricated to demonstrate the possibility to align and interconnect 3 microfluidics levels in one single chip. Scale bars: 500 µm.

Alignment of micro- and macro-molds is very important to make proper interconnects between the channels fabricated with different techniques and then to guarantee conservation of flow and proper functioning of the chip. It is also important to avoid possible leaks or contamination. First, the alignment had to be ensured by the design of the different structures and molds (Figure 4). Then, the positive PDMS molds were cut to the desired size, leaving at least 5 mm of separation from the channels to the edge to avoid possible cracks when connecting luers, reservoirs, or pipette tips. The surface of the PDMS slabs were treated either with plasma etching (PE25-JW, PlasmaEtch inc., Carson, NV, USA) or a homemade corona discharge in order to expose the hydroxyl groups and bond them together. The PDMS layers were then always handled facing down to avoid adhering dust or any other particle to the surface, comprising the adhesion between the different faces of the chips. Using a stereoscopic microscope, the coupling of the micro- and macrostructures were finally aligned and bonded before placing the chip at 90 °C on a hotplate for 30 min and cooling it down slowly to room temperature. For 2-level chips (Figure 4a), the open surface where the channels for micro–macro interconnects were located was easy to align even without any reference marks. In the case of structures with 3 levels or more (Figure 4b), it was necessary to design and place alignment marks to ensure the correct positioning between the different layers. The above procedure is carried out for the two lower layers, making sure to make the holes in the middle layer that connect the upper layer to the lower layer before joining it, Figure 4b. Subsequently, the third layer is attached in the same way with the other two layers, following the alignment marks and with the help of the stereoscopic microscope (SMZ 745T, Nikon Instruments Inc., Melville, NY, USA). Hole punching (EMS-Core, Electron Microscopy Sciences, Hatfield, PA, USA) can be made at any moment of this process although it is easier at the very end of the procedure.

## 3. Results and Discussion

### 3.1. Microfluidic Platforms

#### 3.1.1. Multi-Mold Microfluidic Chips

Several designs were achieved in order to test the multi-mold process with several combinations of macro- and micromold (Figure 4). The 2-level chips tested in this work all succeeded in replicating both the low and high-resolution features in PDMS and the chips were then punched and successfully tested for flows. We also fabricated three microfluidic levels in one single chip for the fabrication of a complex platform to produce microdroplets for several applications like drug delivery and lab- on- chip assays: indeed, this type of chip required multiple inlets and outlets, with some inside existing layers, that could not be achieved with only two molds/layers [24,25,26]. Table 2 shows a practical comparison between single- and multi-mold methods.

#### 3.1.2. Single-Mold Microfluidic Chips

In order to fabricate single-mold chips and thus reduce the cost and fabrication time, we followed the procedure depicted in Figure 3. Laser direct-writing was implemented using our blu- ray system [15] in order to fabricate the macro-regions of a mold for further use in 2PP. In this case, it was important to select carefully a low-resolution resin (the photosensitive material for the low-resolution mold) that would be compatible with the high-resolution process: (1) first, the refractive index mismatch between the cured low-resolution resin and the uncured resins used in the 2PP process had to be sufficient for the 2PP laser to be precisely focused at the interface between the two materials; (2) then, the low-resolution resin had to comply with laser exposure during 2PP and not burn or explode under such radiation; (3) and finally, as the high-resolution resin for the 2PP process has to be developed and cleaned using special chemicals (Figure 3b), it was important that the structures in the first low-resolution resins were not impaired during the second development step. The different photoresins that were tested with our laser direct-writing system either did not enable laser focusing but did burn under 2PP laser exposure, or suffered structural or mechanical modifications during the 2PP development process, especially after a few minutes of immersion. The low-cost commercial Loctite 3525 curable glue compatible with additive manufacturing using our blu-ray system and reported in ref. [15] was found to be an excellent material for the single mold fabrication presented in Figure 3. Loctite 3525 was developed in acetone after laser direct-writing, presents a refractive index of 1.51 which is greater than that of Nanoscribe proprietary resins in their liquid form used in this work (1.486), and it resisted the development step of the 2PP resins used in this work if development lasted less than 15 min. Although this single mold fabrication process was successfully achieved (Figure 3b), the total area that is patternable using laser direct-writing with our system was limited and large areas usually took much more time, compared with CNC micromilling of hard substrates. Although it offers excellent control of the macro- and micro-patterns in a relatively simple manner, for the particular case of large microfluidic chips we preferred the CNC micromilling solution. Interestingly, for other types of applications such as the microfabrication of optical waveguides where the combination of macro- and microstructures are required [27], both cured refractive indices are 1.51.

**Figure 5:**
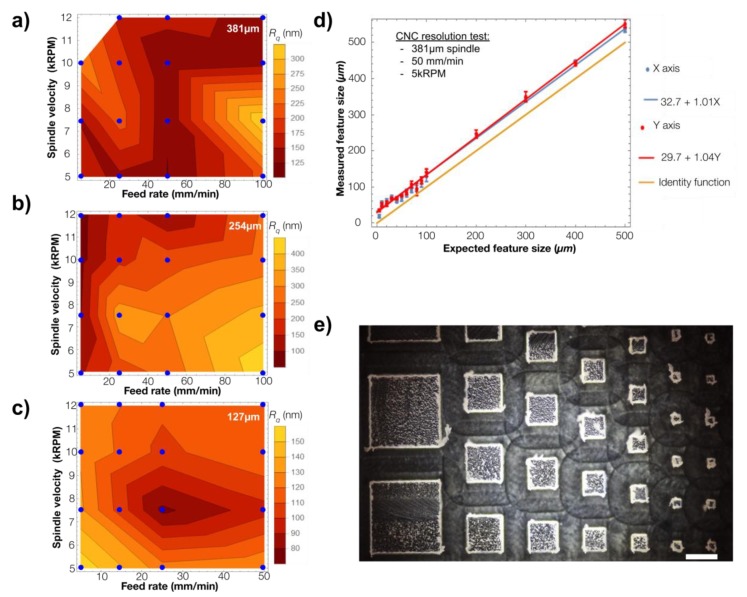
(**a**–**c**) CNC micromilling roughness tests using 3 different end-mill diameters, 381 µm, 254 µm, 127 µm, respectively; the *R_q_* roughness is plotted as a function of both the feed rate and the spindle velocity, the blue points represent our experimental data. (**d**) Comparison between the designed dimension of the features in the resolution test sample and its measurement, the blue line shows the linear fit made to X-Axis data and in red the linear fit of the Y-Axis data, for comparison in yellow the identity function is plotted, each measurement was repeated 6 times, scale bars represent the standard deviation. (**e**) Sample of the out-of-plane square pillars used for the resolutions test. Scale bar is 500 µm.

The preparation of PMMA molds using CNC micromilling is very reproducible and may be used either for micromachining carved features (depths) or out-of-plane structures for mold fabrication. Although the resolution is different for each of the structure types, it is important to test two critical parameters that are dependent on the system used to micromachine the surface of the polymer substrates on which the 2PP laser is then focused to transfer the micropatterns: roughness and resolution [21]. The carved channels are usually the most limiting features in size for CNC micromilling, and as this type of mold was then processed with 2PP where laser focusing is critical, in this work we tested the roughness and resolution of carved structures obtained with our CNC system with different end mill diameters (127 µm, 254 µm, and 381 µm) and speeds (in kRPM) at different feed rates to reproduce a set of flat rectangular structures at a constant depth in order to assess the resulted roughness after the process in flat PMMA molds by performing measurements using a profilometer (KLA Tencor D600, Milpitas, CA, USA). After milling, the samples were placed in a closed reservoir with acetone vapor at 40 °C for 90 s to smoothen the surface. The final roughness was calculated as $R_{q}=\sqrt{\frac{1}{n}\sum_{i=1}^{n}y_{i}^{2}}$ and the results are presented in Figure 5a. Although we found that the roughness may improve with smaller end-mill sizes, it only did very slightly and always at lower feed rates. This would considerably slow the process for large areas and thus limit the rapid prototyping of our molds. Moreover, it is usually recommended to use the most robust tips in solid, stiff materials such as PMMA and the largest end-mill that guarantees a low roughness is often selected. We decided to set the optimized micromilling conditions as follows: 381 µm end-mill, at 50 mm/min and 5 kRPM. When an even faster feed-rate is desired (500 mm/min) we used an end- mill of 3.17 mm at a spindle speed of 10 kRPM. With this configuration a macromold can be milled in less than 5 min with the trade-off of the surface roughness which can be around 300 nm, this value can be further improved with a post-treatment of acetone vapor at 40 °C for 90 s. 

After setting the CNC milling conditions for the best roughness and robustness with the conditions mentioned above, we tested the in-plane resolution of PMMA etching for squares of several dimensions from 5 µm to 500 µm with depths ranging from 5 µm to 100 µm. Figure 5b shows the results of the transferred feature size measured by profilometry against the expected (designed) feature size for all the patterns. As can be seen, our system reproduced the features in both X and Y axes with great fidelity, although an offset of approximately 30 µm was found in each direction. This shows that the CNC micromilling technique is an excellent process for our low-resolution (above 30 µm) mold fabrication and that a high-resolution technique is required for lower resolution. As the PMMA substrates are compatible with the 2PP technique using the Nanoscribe PPGT system, we then used this type of mask for our micro–macro process. 

### 3.2. Biochip Platforms

**Figure 6:**
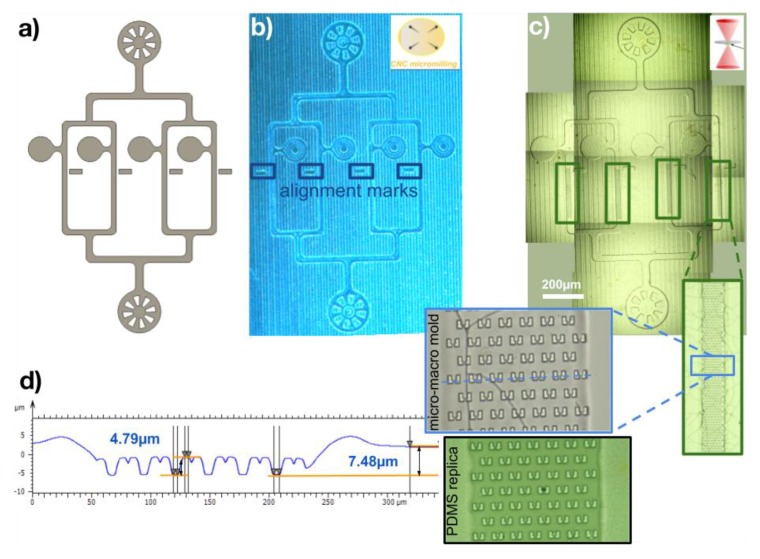
Fabrication process of a master mold for a microfluidic platform replicating an existing one for yeast trapping and study [28]. From a full design including both low- and high-resolution features (**a**), a PMMA sample is first processed using CNC micromilling (**b**) and then transferred to the 2PP system for the fabrication of the microtraps locally, inside a designated small-area of the microchannels (**c**). Although PMMA cleaning with alcohol may generate internal cracks, it was found that the cracks were not transferred in any area of the PDMS replica. (**d**) The surface profile of the PDMS surface, shows the integrity of the microtraps on the replica.

In order to further prove that our technique was robust to fabricate designs that would normally require photolithographic molds for both large (macro) and small (micro) features in bioplatforms, we designed and fabricated two micro–macro molds using a single mold processed first with CNC and then transferred high resolution microstructures using the PPGT. The first design was made for further construction and use of a PDMS microfluidic chip for yeast trapping and study (the study itself is outside the scope of this work and will be reported elsewhere). The design replicated an existing one made with a different process [28] and is presented in Figure 6. As can be seen, after the full design was made, the CNC process transferred the low-resolution channels into a PMMA mold that was subsequently cleaned and placed inside the 2PP system to transfer the microtraps inside the desired channels that had been marked with small, non-intrusive alignment marks outside them. A manual approach had to be made inside the channels as the low-resolution channels needed to be less than 10 µm deep (7.48 µm was measured by profilometry). After carefully developing and rinsing the substrate, IPA created some small non-superficial cracks in the mold (apparent in Figure 6); however, the profilometry and PDMS replicas proved that they did not affect the integrity of the structures as they did not appear at the surface. The process limited the area of the microtraps to a minimum, thus lowering the cost and time of fabrication of the high-resolution features.

**Figure 7:**
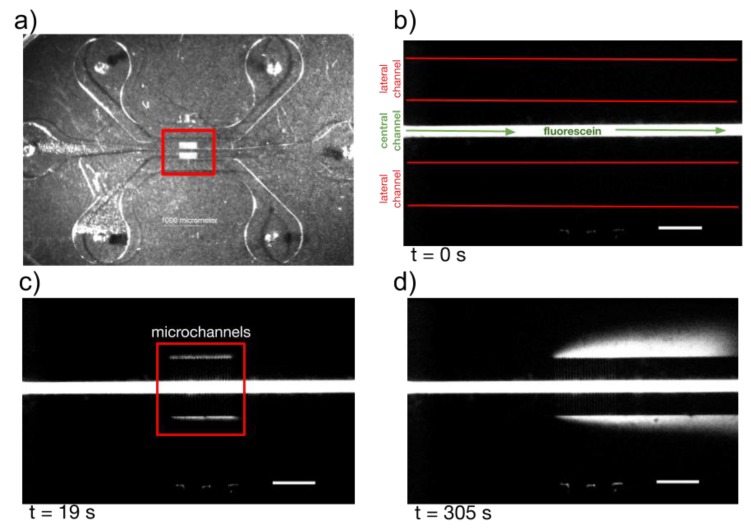
Test of diffusion inside the three-channel microfluidic chip. (**a**) The low-resolution mold for three large channels was made by CNC micromilling (outer channels marked with red lines, central filled with fluorescein in (**b**), the micromold of the smallest connecting microchannels was microfabricated by 2-photon polymerization. (**b**) The channels were first filled with IPA and at time zero, a droplet of fluorescein was deposited at the inlet of the central channel. (**c**) After 19 s, without flow, the fluorescein started to diffuse towards the lateral channels through the connecting microchannels. (**d**) 5 min later, the fluorescein was visible inside the lateral external channels (**d**). Scale bars are all 800 µm.

**Figure 8:**
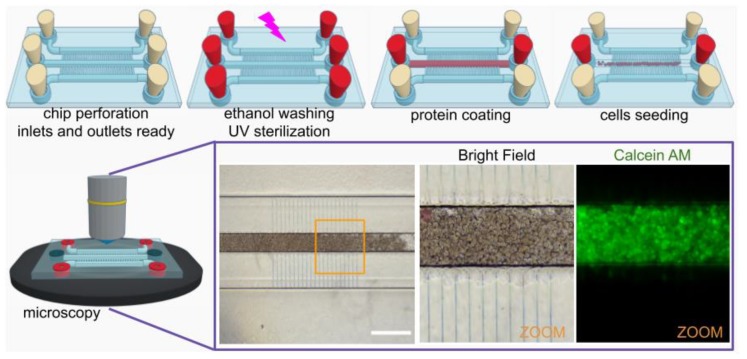
Culture of HepG2 cells inside the central channel with nutrients diffusing from side channels through the lateral high-resolution microchannels. The images were captured with a fluorescence microscope for Calcein AM stained HepG2. Scale bar: 500 µm.

For the second mold and in order to validate the use of the multiresolution fabrication technique presented in this work, HepG2 cells (ATCC, Manassas, VA, USA) were cultured in the central channel of a three-channel chip (Figure 7 and Figure 8). It is known that multiresolution in microfluidics is essential for mimicking the in vivo microenvironment that is needed for the correct development of in vitro models. Hepatic cells are very sensitive to native microarchitecture and blood flow, thus highlighting the relevance of integrate multiresolution in microfluidics for spatial confinement and nutrient diffusion without applying shear stress on cultured cells. A three-channel microfluidic chip, similar to that of Figure 4a, was designed and successfully fabricated for proof-of-concept. In this design, fabricated using the single-mold technique, a central microfluidic channel 200 microns wide is connected to two lateral 1 millimeter wide channels by much smaller microchannels (500 µm (L) × 10 µm (W) × 3 µm (H)). The three large channels were fabricated for the low resolution part of the mold, this channels are designed to confine the cells (central channel) and transport the nutrients (lateral channels). The microchannels (high resolution part of the mold) are used for single-cell migration or nutrient diffusion without applying shear stress on cells cultured in the main central channel [29,30]. After the CNC micromilling mold was made and inspected, the mold was cleaned and the microchannels were successfully transferred to the existing mold following the steps of the 2PP process described in a previous section. After inspection of the final mold, a PDMS replica was obtained using REM, the chip was sealed and the diffusive function of the microchannels fabricated with 2PP was successfully tested using fluorescein inside the central channel and observing fluorescence inside lateral channels (Figure 7). 

Finally, the microchannels were sterilized with three 70% ethanol washes and followed by 30 min UV exposure (λ = 253.7 nm). In order to promote the cell adhesion to the channels a 1 mg/mL collagen I (Corning Inc., Corning, NY, USA) coating was performed and incubated for 2 h before seeding. Collagen was replaced with culture medium: MEM + 10% FBS and 1% penicillin-streptomycin (all from Gibco, Waltham, MA, USA). Then, 200 µL of concentrated HepG2 cells suspension was injected in the central channel, filling it by diffusion. Before the cells were introduced, they were incubated for 15 min in a 1 µmol/L calcein AM (Invitrogen, Waltham, MA, USA) medium and observed after the medium of the channel was changed, 2 h after they were introduced. The fluorescence emitted by activated calcein was elected as a signal of cell viability (Figure 8). The fluorescence was observed until 24 h later, at the end of this experiment, only used as a proof-of-concept test to prove the compatibility of our technique with cell culture protocols such as sterilization, protein coating to increase the cell adhesion, cell seeding, and cell imaging inside the channels. On further biological applications, other conditions such as flow rate will be studied.

## 4. Conclusions

We showed that it is possible to fabricate microfluidics devices integrating expensive micron-range features with a simple rapid-prototyping technique by separating the costly master molds containing the high-resolution (“micro”) patterns from low-resolution molds with the generic larger (“macro”) channels and connection ports. While common photolithographic strategies were used for the high-resolution parts of the designs, several simpler options were successfully tested for the interconnects, such as CD-DVD-Bluray laser micromachining, micromilling and polystyrene sheet-controlled shrinkage. It was possible to merge both designs in one PDMS chip by simple alignment and assembly of two or more microfluidic levels. It was also possible to transfer the micropatterns directly onto an existing macromold by using a 2-photon polymerization technique thus obtaining a single mold for ease of use in replica molding. We believe this micro–macro process presented here is an excellent solution for small-budget laboratories as it limits greatly the cost and time of fabrication or outsourcing of a limited area containing expensive micropatterns. 

## Figures and Tables

**Table 1:** Comparison of the resolution, area and speed of the different fabrication methods used in this work. The reported fabrication speed is calculated with the different fabrication parameters used for each technique.

Fabrication Method	Minimum Feature Size	Maximum Fabrication Area	Fabrication Speed
**2PP**	300 nm	3 × 3 × 2 mm	99–6000 min/mm^3^
**Direct laser writing**	10–20 µm	25 × 25 mm	5–500 min/mm^3^
**CNC micromilling**	127 µm	150 × 150 × 150 mm	0.37–202 min/mm^3^
**Shrinky Dinks® molding**	200 µm	⅓ of an A4 sheet	0.02–1 min/mm^3^

2PP—2-photon laser polymerization; CNC—Computer Numerical Control.

**Table 2:** Comparison between single- and multi-mold methods in terms of flexibility, need of alignment. and compatible fabrication methods.

Method of Integration	Compatible “Macro” Fabrication Methods	Need for Alignment	Alignment Tolerance	Flexibility
**Single-mold**	CNC micromilling	Once with the fabrication of the mold	Up to 10 µm	Only works with one design
**Multi-mold**	Direct laser writing	Every chip	Up to 300 µm	One “macro” design can be used with several “micro” designs
	CNC micromilling			
	Shrinky Dinks® molding			
